# Anomalous experiences, psi and functional neuroimaging

**DOI:** 10.3389/fnhum.2013.00893

**Published:** 2013-12-23

**Authors:** David J. Acunzo, Renaud Evrard, Thomas Rabeyron

**Affiliations:** ^1^School of Informatics, Institute for Adaptive and Neural Computation, University of EdinburghUK; ^2^SULISOM, Psychology Department, University of StrasbourgStrasbourg, France; ^3^LPPL, Psychology Department, University of NantesNantes, France

**Keywords:** anomalous experiences, telepathy, precognition, distant intentionality, fMRI, psi, methodology

Over the past decade, there has been increasing scientific interest in anomalous experiences. These can be defined as “uncommon experience[s] […] that, although [they] may be experienced by a significant number of persons […], [are] believed to deviate from ordinary experience or from the usually accepted explanation of reality according to Western mainstream science” (Cardeña et al., 2014). This scientific interest has led to important contributions toward the understanding of several aspects of these experiences (Brugger and Mohr, 2008). One of the most controversial hypotheses associated with anomalous experiences is the *psi hypothesis*, which states that anomalous experiences sometimes imply forms of interactions falling outside currently known biological and physical mechanisms (Bem and Honorton, 1994). Thus, far, small but persistent effects are frequently reported in experiments testing the psi hypothesis (Radin, 2006), while no consensus has been reached concerning their explanation (Alcock et al., 2003).

Research testing the psi hypothesis has occasionally generated a great deal of interest and controversy. The most recent example is Bem's series of precognition experiments (Bem, 2011), which triggered important methodological questionings on the validity of the frequentist approach (Miller, 2011; Rouder and Morey, 2011; Wagenmakers et al., 2011), widely used in experimental sciences. Bem's paper was followed by an attempt of replication (Ritchie et al., 2012a), which resulted in reflections on the difficulty in publishing direct replications in psychology (Ritchie et al., 2012b). This debate, still ongoing, has shown how research about anomalous experiences can stimulate cutting-edge discussions on scientific methodology. This heuristic value of anomalous experiences has a history even in the infancy of cognitive neuroscience with the German neurologist Hans Berger, inventor of electroencephalography and the first person to describe different brain waves, having previously had a telepathic experience with his sister which made him obsessed by the idea of how his mind could have carried such a signal (Berger, 1940).

More recently, attempts to test the psi hypothesis and find its neural correlates have been carried out using functional neuroimaging. The rationale behind these experiments is that if psi-related processes are indeed present in the brain, even unconsciously, they should be observable using functional neuroimaging. An example of such a study would be to test whether the brain activity of Participant A would be influenced when Participant B, situated in another isolated room, intends to send information to or simply concentrate on Participant A. Various types of hypothetical phenomena have already been examined, including forms of telepathy (Standish et al., 2003; Richards et al., 2005; Moulton and Kosslyn, 2008; Venkatasubramanian et al., 2008), distant intentionality (Achterberg et al., 2005), and precognition (Bierman and Scholte, 2002; Moulton and Kosslyn, 2008). All these six studies but one (Moulton and Kosslyn, 2008) reported results consistent with the psi hypothesis.

Unfortunately, several of these studies suffer from methodological weaknesses that could account for the reported effects. Listing these flaws may contribute to the improvement of the research in this field. These methodological weaknesses can be grouped into four categories:

1. *Counter-balancing* across participants is routinely used in experimental psychology and cognitive sciences to avoid systematic biases due to experimental conditions specific to one or several participants. In Venkatasubramanian et al. (2008), the receiver and the sender were presented green and red-colored stars to indicate the onset of telepathy and control trials, respectively. It is therefore not possible to know whether the difference in brain activity between the two conditions is due to the nature of the trial (telepathy vs. control) or to the difference in the color of the stimulus indicating trial onset. To disentangle this potential confound, the reverse cue association has to be given for half of the trials—or half of the participants, if their number is sufficient.
2. *Trial order randomization* prevents biases that could be caused by the particular order of the trial conditions. Such biases can be caused by participants detecting a certain pattern (e.g., repetitions or alternations), leading to expectations and thus detectable neural signatures that could bias the results. Habituation, leading to different brain activity between the beginning and the end of the experiment may also bias the results. To counter-balance the potential biases produced by a particular sequence—even if it was generated randomly—each participant should be given a distinct series of randomly-ordered trials. Unfortunately, proper randomization was not met in four of the six studies: In Venkatasubramanian et al. (2008) no randomization was used at all, while in Standish et al. (2003) and Richards et al. (2005) the duration of the trials was randomized, but not their order. Moreover, in the Venkatasubramanian study, the target picture was freely chosen and drawn with a pen by one investigator used as the “sender.” A randomized target selection from a prepared set of images would have been preferable. Humans are indeed inherently biased in their attempts to generate random targets (Brugger and Taylor, 2003). Besides, a randomization would have prevented potential correlations between the target imagined by the “sender” and the guess of the “receiver” due to their potential interaction or common immediate past experience before the experiment.
3. *Information shielding*: All normal mechanisms have to be excluded for correlations between the source (e.g., a “sender” or healer) and the participant's brain activity to be considered as psi (see e.g., Alcock et al., 2003). However, some reports showed weaknesses on this crucial point. In Achterberg et al. (2005), the healer's task is to influence from a distance the participant lying inside the scanner. In this study, the same healer was used for three different participants while the same sequence (i.e., the order of control and active sessions) was used. Consequently, this particular healer knew in advance this sequence, and it is not specified whether contact between the healer and the participants was prevented. In the Venkatasubramanian et al. (2008) study, the authors used the same target image for the only two participants whilst no information concerning a possible interaction between them was provided, potentially leading to the same problem.
4. *Small sample size*, i.e., too small a number of participants and/or trials per participant, was also a weakness of several studies (Standish et al., 2003; Richards et al., 2005; Venkatasubramanian et al., 2008). As underpowered studies most often miss existing effects (leading to false negatives), reported positive results have a low probability to reflect a true effect (see e.g., Button et al., 2013). Furthermore, with too few participants, proper counter-balancing is difficult and the risk of confounds is greater.

Two studies (Bierman and Scholte, 2002; Moulton and Kosslyn, 2008), however, appear methodologically sound. Both explored various potential sources of artifacts that could account for their respective significant results. Bierman and Scholte (2002) could not find any classical explanation for the significant effects observed. Moulton and Kosslyn (2008), on the other hand, concluded that their results constituted “the strongest evidence yet obtained against the existence of (psi)” despite the logical difficulties in proving a negative existential proposition (Whitehead and Russell, 1910–1913). Additionally, despite the many precautions taken by the experimenters, a subtle bias was still found in one participant's data, indicating that the design could potentially be flawed.

Finally, none of the studies addressed the issue of the confined and noisy environment inside the scanner tube that tends to make participants uncomfortable. As this problem is currently unavoidable, the participants could be prompted about their comfort or relaxation level and their answers used as a covariate in the analysis.

Testing the psi hypothesis using neuroimaging is an important topic as it may help to shed some light on the nature of anomalous experiences (Watt and Irwin, 2010; Krippner and Friedman, 2010b), on altered states of consciousness (Krippner and Friedman, 2010a; Cardeña and Winkelman, 2011) and more generally on potential methodological problems in the field of psychology and neurosciences (Watt, 2005). Nevertheless, in our opinion, no firm conclusions concerning the psi hypothesis can be made on the basis of this corpus of functional neuroimaging data, and more methodologically sound results need to be generated.

## Author contributions

All authors contributed extensively to the work presented in this paper.
